# Nutritional Status, Social Determinants of Health and Clinical Outcomes in Critically Ill Children

**DOI:** 10.3390/children12030390

**Published:** 2025-03-20

**Authors:** Yash Desai, Andrea Marroquín, Paola Hong-Zhu, Nicole Knebusch, Stephanie Vazquez, Marwa Mansour, Thomas P. Fogarty, M. Hossein Tcharmtchi, Fernando Stein, Jorge A. Coss-Bu

**Affiliations:** 1 McGovern Medical School, UTHealth, Houston, TX 77030, USA; yash.m.desai@uth.tmc.edu; 2 Division of Critical Care, Department of Pediatrics, Baylor College of Medicine and Texas Children’s Hospital, Houston, TX 77030, USA; 3 Department of Pediatrics, Texas Tech University Health Sciences Center, Lubbock, TX 79430, USA

**Keywords:** malnutrition, socioeconomic status, critically ill, pediatrics, intensive care, outcomes

## Abstract

Introduction: Childhood malnutrition remains a risk factor for morbidity and mortality. Children admitted to the Pediatric Intensive Care Unit (PICU) are at a higher risk of worsening nutritional status with adverse clinical outcomes. The burden of malnutrition is strongly linked to various well-defined social determinants of health, including race, socioeconomic status, and geography, as these factors influence household food insecurity. This study aimed to analyze the interrelationships of nutritional status, social determinants of health, and health outcomes in critically ill children. Methods: Retrospective cohort study of 6418 critically ill children admitted to PICU from January 2014 to December 2017. Demographic and anthropometric measurements were collected upon admission and outcomes. Based on the patient’s zip code, and median household income, we estimated the percentage of the population living in poverty, and the percentage of the population experiencing food insecurity for 5912 children. Results: The prevalence of underweight, chronic, and acute malnutrition was 13.2%, 17.9%, and 5.6%, respectively. Malnourished children had longer duration of mechanical ventilation and longer PICU and hospital lengths of stay (LOS) compared to nourished children. Underweight and chronic malnutrition were associated with higher mortality. Hispanic children had the highest prevalence of poverty level, while non-Hispanic Black children had the highest food insecurity level and lowest median income. Ethnicity was not associated with mortality. Conclusions: Malnourished critically ill children who were disproportionately non-Hispanic Black, Hispanic, and Asian had worse hospital outcomes, including prolonged hospital and PICU length of stay, increased time on mechanical ventilation, and a higher risk of mortality.

## 1. Introduction

Critical illness elevates metabolic demands during the initial phases of the stress response. However, meeting these nutritional demands is often challenging. The combination of increased requirements and inadequate substrate intake contributes to poor clinical outcomes, especially in malnourished children [1,2,3].

Thus, children admitted to the PICU are at a higher risk of worsening nutritional status and anthropometric changes, both of which could correlate with adverse clinical outcomes. Childhood malnutrition is a leading topic in the medical arena and public health landscape as a threat to illness and mortality. In all forms, childhood malnutrition (underweight: weight for age, chronic malnutrition: height for age, and acute malnutrition: weight for length) is a significant barrier to achieving critical developmental milestones in physical, emotional, and cognitive health [4,5]. Furthermore, this pathology is associated with increased infectious and hereditary disease (Diabetes, cardiovascular disease, cancer) risk [5,6]. We believe that this burden of childhood malnutrition extends into the clinical setting as well and is linked to bad clinical results in sick children.

The social determinants of health (SDH) refer to a series of external (environmental, political, geographical, economic, etc.) factors that change a person’s health and well-being [7]. Important examples of social determinants of health include race, socioeconomic status, neighborhood location, access to employment opportunities, education, and healthcare [7]. In recent years, medical academia has begun acknowledging the interplay between sociological factors and patients’ overall health. Understanding this interplay is of utmost importance, as it shifts the treatment paradigm in a way that recognizes the multidimensional and multidisciplinary aspect of healthcare. This informs healthcare decision-making by emphasizing the need to address relevant social factors when providing holistic patient care. The significance of SDH cannot be understated. In the United States alone, over 100,000 deaths per year are linked to poor SDH, many of which can be overcome through appropriate resource allocation [7]. Disproportionately, these adverse social determinants of health affect racial and ethnic minorities, resulting in poorer health outcomes among said groups in comparison to their white counterparts [7].

The burden of malnutrition is strongly linked to various well-defined social determinants of health, including race, socioeconomic status, and geography, as these factors influence levels of household food insecurity [7,8,9]. Food insecurity is a socioeconomic condition in which consistent and affordable access to adequate, quality, and nutritious food is either unreliable or uncertain [9]. Specifically, delineating the qualifying factors “quality” and “nutritious” are of extreme relevance, as they allow childhood obesity (where children may have access to food itself, but not quality or nutritious food) to fall under the category of malnutrition as a variable that requires similar future consideration. Of particular interest in this study, low household income is believed to be a key hazard factor for food insecurity, not only due to the consistent inability to afford healthy, quality whole foods but also due to the geographic placement of lower-income communities, which are often located far away from grocery stores [7]. Altogether, childhood poverty and its associated outcomes fall under the category of adverse childhood experiences, which expose children to significant toxic stresses that influence health outcomes [7].

Addressing food insecurity and understanding malnutrition and its consequent health outcomes should be an essential mission of public healthcare, given the prevalence of these social and medical pathologies. As per the WHO in 2020, among children under the age of 5, global rates of malnutrition were as follows: 149 million children suffered from chronic malnutrition (low height for age), 45 million were affected with acute malnutrition (low weight for height), and 38.9 million children suffered from overweight and obesity [10,11]. In the United States in 2020, around 15% of American households experienced some form of food insecurity [12,13], and 35.3% of these households were below the U.S. federal poverty line [12,13]. As mentioned, disparities regarding food insecurity and malnutrition are present along racial/ethnic lines. Twenty percent of African American households and 16% of Hispanic/Latino households experienced some level of food insecurity at some point in 2021, compared to 7% of White households [12,13].

To further analyze the interrelationships of nutritional status, social determinants of health, and corresponding health outcomes in critically ill children, the aims of this study are:

1. To analyze malnutrition and its associated clinical outcomes in critically ill pediatric patients.
2. To investigate the relationship between patient ethnicity, malnutrition, and adverse clinical outcomes.
3. To explore the connection between ethnicity, socioeconomic factors (such as median income and poverty rates), and food insecurity.

## 2. Materials and Methods

A retrospective cohort study of 6418 critically ill children admitted to the Pediatric Intensive Care Unit (PICU) at Texas Children’s Hospital from January 2014 to December 2017. The Baylor College of Medicine Institutional Review Board approved the study H-39403 on 17 March 2021.

This study obtained data from the electronic medical record (EMR), including demographic and anthropometric measurements upon admission, length of stay (LOS) in the PICU and hospital LOS, primary diagnosis, and time spent on mechanical ventilation (MV). The risk of mortality (ROM%) and the Pediatric Risk of Mortality (PRISM) Score III were calculated on admission. The patient’s insurance category was classified as government-paid or non-government.

On admission to the PICU, the recumbent length of children younger than 24 months was measured by using a length board. For older children who were unable to stand, knee/heel measurements were obtained by using knee/heel calipers and were converted to height as reported earlier [14] Weight was obtained with digital scales (Scale-Tronix Model 2309, Scale-Tronix Inc., Ann Arbor, MI, USA) and digital baby scales (Olympic Smart Scale, Model 56350, Olympic Medical, Seattle, WA, USA). Each patient’s anthropometric parameters (height and weight) were compared to growth charts at a given age and gender, as per WHO for children from 0 to 24 months and CDC for children from 2 to 19 years [15]. Nutritional status was defined based on a patient’s weight for age (WFA; underweight), height for age (HFA; chronic malnutrition), and weight for length (WFL/BMI) z-score as this corresponded to WHO/CDC data. Within each category, malnutrition was defined as z-score < −2. Other patient data, including ethnic group and zip code, were obtained from patient charts. Zip code data offered insight into metrics, including the zip code’s median household income, the percentage of the population living in poverty, and the rate of the population experiencing food insecurity, based on data compiled by the Houston Food Bank in 2017 (https://public.tableau.com/app/profile/valerie.hawthorne/viz/ZipCodeData_0/ZipCodeData accessed on 1 May 2024) [16]. Given that a patient’s socioeconomic and food security status could not be identified directly, the data provided by the patient’s zip code were used as a proxy to assess these factors for the analysis. Classifications of income levels into categories of low (median income < 45,200 USD), middle (median income of 45,200–135,600 USD), and upper income (median income > 135,600 USD) were obtained from parameters set by the Pew Research Center in 2018 (https://www.pewresearch.org accessed on 1 May 2024) [17]. Low-income communities are categorized as having a median income. Patients without a documented zip code were excluded from this dataset. A total of 5912 children were included in this analysis.

The descriptive data are shown as averages with standard deviation (SD) or medians with interquartile range (IQR. 25th–75th) for continuous variables and frequencies with percentages for categorical variables. The non-parametric data were evaluated using the Mann–Whitney and Fisher exact tests to compare continuous and categorical variables. The Kruskal–Wallis test was applied to compare more than two groups. Statistical significance was determined as *p* < 0.05. Statistical analysis was completed with Stat View Version 5.0.1 (SAS Institute Inc., Cary, NC, USA).

## 3. Results

A total of 6418 patients were identified and entered into the analysis, and 44% were females. Most patients (87%, n = 5575) had a medical diagnosis, while the remaining were surgical patients. Additionally, 49.9% of the children had a chronic medical diagnosis. For the total group of children, the median and interquartile (IQR) for age was 4.77 (1–12) years, the Pediatric Risk of Mortality III (PRISM III) score was 3 (0–7), risk of mortality (ROM) was 0.63 (0.30–1.64)%, the duration of MV (n = 2379) was 74 (23–168) hours, PICU LOS was 1.80 (0.90–4.20) days, hospital LOS was 7.85 (3.73–16.3) days, and hospital mortality was 3.29%, (n = 211) (Table 1).

Regarding the anthropometric evaluation, the weight was 17.3 (9.2–41.4) kg., the height was 104.7 (74–145) cm, the weight for age z score was −0.23 ± 1.90 (SD), the height for age z score was −0.49 ± 1.91, and the weight for length z score was −0.06 ± 1.22. The prevalence of underweight, chronic, and acute malnutrition was 13.2%, 17.9%, and 5.6%, respectively (Table 2).

### 3.1. Nutritional Status, Ethnicity, and Clinical Outcomes

The distribution of patients with underweight (n = 847) based on ethnicity was non-Hispanic Black: 19.48% (n = 165), Asian: 6.61% (n = 56), Hispanic: 44.39% (n = 376), non-Hispanic white: 27.15% (n = 230), and other: 2.36% (n = 20), χ 9.55, *p* = 0.0486. The distribution of patients with chronic malnutrition (n = 1151) based on ethnicity was non-Hispanic Black: 16.59% (n = 191), Asian: 5.91% (n = 68), Hispanic: 46.48% (n = 535), non-Hispanic white: 28.84% (n = 332), and other: 2.17% (n = 25), χ 22.32, *p* = 0.0002. The distribution of patients with acute malnutrition (n = 360) based on ethnicity was non-Hispanic Black: 23.33% (n = 84), Asian: 6.67% (n = 24), Hispanic: 38.61% (n = 139), non-Hispanic white: 27.22% (n = 98), and other: 4.17% (n = 15), χ 8.94, *p* = 0.0627.

The relationship relating malnutrition and risk of mortality evaluated by PRISM III showed a ROM for underweight vs. non-underweight children of 0.80 (0.38–2.21)% vs. 0.57 (0.30–1.64)%, *p* < 0.0001; while the ROM for children with chronic malnutrition vs. non-chronic malnourished children was 0.79 (0.31–2.01)% vs. 0.52 (0.30–1.37)%, *p* < 0.0001, there was no significant difference in ROM between children with acute malnutrition vs. children without. For each type of malnutrition, underweight, chronic, and acute malnutrition, the malnourished group of children had a longer duration of mechanical ventilation, PICU, and hospital length of stay than the non-malnourished group (Table 3).

The relationship between malnutrition and hospital mortality showed a higher likelihood of death only for children with underweight and chronic malnutrition, with no association between acute malnutrition and mortality (Table 4).

The clinical outcomes, socioeconomic indicators, and their corresponding ethnicity are shown in Table 5.

### 3.2. Insurance Category, Ethnicity, Income, and Food Insecurity

The distribution of patients (n = 4222) on government insurance by ethnicity was non-Hispanic Black: 24.16% (n = 1020), Asian: 3.06% (n = 129), Hispanic: 52.18% (n = 2203), non-Hispanic white: 18.17% (n = 767), and other: 2.44% (n = 103), χ 962.1, *p* < 0.0001. The median income/yr in thousands of USD, percentage for poverty level, and percentage of food insecurity in children with government insurance compared to non-government insurance were 43.9 (36–59) vs. 64.7 (45–87), *p* < 0.0001; 20.7 (13.2–28.8) vs. 11.7 (6.3–18.3), *p* < 0.0001; and 18.8 (15.5–24) vs. 16.1 (13.9–19.2), *p* < 0.0001, respectively (Table 6).

The allocation of income categories in children with government insurance for low, middle, and high was 33.15%, 51.42%, and 15.43%, respectively. The percentage of food insecurity based on income category for low (n = 1501), middle (n = 2956), and high (n = 1455) income was 23.4 (18–29), 18.8 (16–22), and 14.3 (13–16), respectively, *p* < 0.0001 (Table 7).

## 4. Discussion

This retrospective investigation showed insight into the interplay between relevant social determinants of health (race, socioeconomic status, and level of food security) and the prevalence of malnutrition and associated clinical outcomes.

The prevalence found in this study for the three categories of malnutrition underweight (13.2%), chronic (17.9%), or acute malnutrition (5.6%) were similar to the reported prevalence in the literature in critically ill children from high-income countries, ranging from 13.4% to 18.6% [18,19,20]. Regarding the impact of undernourishment on critically ill children, several studies have reported adverse clinical outcomes in malnourished children admitted to the PICU [21,22,23]. Our study findings mirror the previous literature reports, with a significantly higher risk of mortality, duration of mechanical ventilatory support, PICU, and hospital LOS in malnourished children for all three malnutrition categories compared to the non-malnourished group.

The whole mortality rate in this cohort of 3.29% was similar to the mortality rate previously published by Kyle U et al. at our institution at 2.9% [24] and lower than the mortality rates published by Valla et al. at 7.7% [19], Bechard et al. at 7% [22], and Delgado et al. at 18.5% [23]. The range of reported mortality rates could be explained based on differences in the patient’s diagnostic category, nutritional status, sepsis, oncologic diagnosis, etc. Our study found a significantly higher mortality associated with underweight and chronic malnutrition with OR of 1.47 (1.02–2.10) and 1.44 (1.04–1.99), respectively; these values are similar to the association between mortality and underweight reported by Bechard et al. [22] with OR of 1.53 (1.24–1.89). The research of de Souza et al. [21] and Delgado et al. [23] did not report an association between mortality and malnutrition.

There was an association between patient race and elements of socioeconomic status. Hispanic and Black children were more likely to come from lower-income communities compared to other studied demographic groups. Consequently, Hispanic and Black patients were more likely to come from communities with higher rates of poverty and food insecurity (Table 4). Patient ethnicity was also associated with the prevalence of HFA, WFA, and WFL malnutrition, with Hispanic and Asian children having disproportionately high rates of HFA and WFA malnutrition. Black patients had disproportionately high rates of WFL malnutrition. Increased severity of HFA, WFA, and WFL malnutrition was associated with higher mortality rates, PICU length of stay, hospital length of stay, and time on mechanical ventilation (Table 3). Hispanic, Black, and Asian parents experienced longer hospital lengths of stay and higher rates of mortality than their White counterparts (Table 4). This study suggests that Hispanic and Black patients are more likely to come from lower-income neighborhoods with higher rates of food insecurity and are consequently expected to present to the hospital with poorer nutritional status and experience worse outcomes.

This last statement is a crucial qualifier in this analysis. Patient race does not act as a social determinant of health in and of itself. Instead, it exists because of systemic social conditions that racial minorities, namely Black and Hispanic minorities, are expected to face, such as income inequality, environmental racism, barriers to healthcare access, and treatment biases [25,26]. As such, Najjar and associates indicate that future studies on social determinants of health ought to examine the broader spectrum of structural racism and its associated factors altogether (e.g., housing disparities, access to a consistent and affordable means of transportation, access to green spaces, industrial zoning, etc.) [26].

Results from other studies have corroborated the social findings of this study. Bush and colleagues conducted international research on SDH and their influence on respiratory health. They found that patients from lower socioeconomic backgrounds (who tended to be racial minorities as opposed to White in high-income countries) were disproportionately burdened by poorer respiratory symptoms and health outcomes [27]. These findings were likely to be due to an amalgamation of factors, including proximity to industrial pollutants and lack of access to affordable medical care [27]. A narrative literature review by Sullivan and colleagues highlighted that children from minority and low-income communities experience disproportionately higher rates of asthma, which is the most common chronic disease among pediatric patients [28]. Similarly, the review concludes that this disparate outcome in disease prevalence is due to the causes mentioned above (pollutant exposure and poor access to medical care) and the effect of poverty as a form of toxic stress on biological regulation [28].

As this study suggests, poverty is a well-documented adverse childhood experience (ACE) that is known to influence health outcomes, primarily due to its association with a series of other ACEs (e.g., housing insecurity, alcohol/tobacco/drug use and exposure, exposure to violence, etc.) [29,30]. An analysis by Rodenbough and colleagues suggests that high early exposure to ACEs can negatively impact a child’s brain development, immune response, and stress response [30]. The implications of these impacts can carry on into adulthood [30]. In their study, Rodenbough and colleagues specifically find that patients admitted to the PICU experience a higher-than-average level of ACE exposure, further highlighting the importance of such studies that seek to understand the link between a multitude of various ACEs and their pediatric outcomes, especially in critically ill children [30]. Chung and colleagues further emphasize that clinicians should screen pediatric patients for food insecurity to better connect them with necessary community resources, such as food banks and free school meal programs [31]. In turn, this could limit the burden of poverty as an ACE on a child’s growth, development, and clinical outcomes [31].

While this study provided a comprehensive understanding of the interplay and the associations between socioeconomic indicators, nutritional status, and clinical outcomes in this large cohort, it was not without its limitations. Primarily, due to the nature of this study as a single-institution retrospective analysis, the discussed results may not necessarily be generalizable to a broader population. Addressing this limitation would involve conducting meta-analyses of similar studies and procuring data from other institutions that are studying similar trends within their patient population. On this note, certain relationships between observed factors were suggested (e.g., Race and hospital LOS; HFA malnutrition and mortality) but were not statistically significant. To further examine these relationships, a study with a higher power is warranted. Moreover, this study incorporates data from 7 to 10 years ago and uses patient’s zip code as a proxy to suggest a patient’s financial background and food insecurity status. Patients whose zip code data were unavailable were not included in the results of the study. Over the past 7–10 years, the median income and food insecurity rate in a given zip code have likely changed. Furthermore, patients coming from low-income communities with higher rates of food insecurity may not necessarily come from a low-income or food-insecure background themselves. Future studies should work to obtain patient-specific income backgrounds and screen each patient for food insecurity using a designated algorithm to account for this limitation.

An interesting finding from the results of this study that ought to be further examined is the rates of malnutrition and poor hospital outcomes among Asian patients. Specifically, this study found that Asian patients experience a prevalence of malnutrition on PICU admission and poor hospital outcomes that are similar to, or worse than, those of Black and Hispanic patients, despite coming from higher-income communities with lower rates of food insecurity. Future studies should work to understand the potential social and systemic factors that account for this.

## 5. Conclusions

This study highlights an interplay between social determinants of health (race, income, food insecurity), nutritional status on PICU admission, and relevant clinical outcomes (hospital/PICU length of stay, risk of mortality, and duration of ventilatory support). Specifically, this study suggests that Black and Hispanic minorities are coming from lower-income communities with higher rates of food insecurity and are more expected to present to the PICU with poor nutritional status. In turn, patients with poor nutritional status (who are disproportionately Black, Hispanic, and Asian) are more likely to experience poor hospital outcomes, including prolonged hospital and PICU length of stay, increased time on ventilatory support, and a higher risk of mortality. This suggests that clinical providers should be more vigilant upon admission to the hospital about a patient’s social determinants of health status and use these data to integrate into their clinical approach and inform patients of their long-term care plans (e.g., connecting patients and families with resources that allow them to have access to health care and obtain quality, nutritious food). Future studies that have a higher power should incorporate data from multiple institutions, and procure patient-specific social data, which are key to addressing the limitations of this study.

## Figures and Tables

**Table 1:** Patient characteristics.

	N = 6418
Age (years)	4.77 (1–12)
Female/Male (%)	44/56
Medical diagnosis n (%)	5575 (86.9)
Surgical diagnosis n (%)	843 (13.1)
Chronic medical diagnosis n (%)	3204 (49.9)
PRISM III score	3 (0–7)
PRISM III Risk of mortality (%)	0.63 (0.30–1.64)
Duration of mechanical ventilation (h) (n = 2379)	74 (23–168)
PICU length of stay (days)	1.80 (0.90–4.20)
Hospital length of stay (days)	7.85 (3.73–16.3)
Mortality n (%)	211 (3.29)

Values are median (IQR; 25th–75th). PRISM: Pediatric Risk of Mortality; PICU: Pediatric intensive care unit.

**Table 2:** Anthropometric evaluation and nutritional status.

	N = 6418
Weight (kg.)	17.3 (9.2–41.4)
Height (cm)	104.7 (74–145)
Weight for age z score	−0.23 ± 1.90 (SD)
Prevalence of underweight n (%)	847 (13.2)
Height for age z score	−0.49 ± 1.91
Prevalence of chronic malnutrition n (%)	1151 (17.9)
Weight for length/BMI for age z score	−0.06 ± 1.22
Prevalence of acute malnutrition n (%)	360 (5.6)

Values are median (IQR; 25th–75th) and average ± SD. BMI: Body mass index.

**Table 3:** Clinical outcomes based on nutritional status.

N = 6418	WFA z Score < −2 N = 847	WFA z Score > −2 N = 5571	HFA z Score < −2 N = 1151	HFA z Score > −2 N = 5267	WFL/BMI z Score < −2 N = 360	WFL/BMI z Score > −2 N = 6058
PRISM III score	4 (1–8) ***	3 (0–7)	4 (0–8) ***	3 (0–7)	3 (0–7)	3 (0–7)
PRISM III ROM (%)	0.80 (0.38–2.21) ***	0.57 (0.30–1.64)	0.79 (0.31–2.01) ***	0.52 (0.30–1.37)	0.63 (0.34–1.79) **	0.63 (0.30–1.64)
Duration of MV (h)	97 (42–212) ***	70 (21–164)	93 (35–198) ***	71 (21–163)	106 (42–196) *	71 (22–166)
PICU LOS (days)	2.2 (1.1–5.5) ***	1.7 (0.88–4)	2.1 (0.99–5.2) ***	1.7 (0.88–3.9)	2.8 (1.2–6.9) ***	1.7 (0.89–4.01)
Hospital LOS (days)	11 (5.6–22) ***	7.4 (3.6–15)	10 (5.2–22) ***	7.3 (3.6–15)	9.7 (4.9–19) ***	7.7 (3.7–16)

Values are median (IQR; 25th–75th). WFA: weight for age; HFA: height for age; WFL/BMI: weight for length/Body mass index; PRISM: Pediatric Risk of Mortality ROM: Risk of mortality; MV: mechanical ventilation; PICU: pediatric intensive care unit; LOS: length of stay. Comparison by Mann–Whitney for each type of malnutrition; * *p* < 0.001, **, *p* < 0.0005, *** *p* < 0.0001.

**Table 4:** Association of nutritional status and mortality.

Type of Malnutrition	Coefficient	Odds Ratio	95% C.I.	*p* Value
Underweight	−3.44	1.47	1.02–2.10	0.0368
Chronic Malnutrition	−3.46	1.44	1.04–1.99	0.0273
Acute Malnutrition	−3.40	1.30	0.76–2.22	0.3370

Analysis by univariate logistic regression; C.I.: confidence interval. Underweight is defined as weight for age z score < −2, chronic malnutrition is defined as height for age z score < −2, and acute malnutrition is defined as weight for length z score < −2.

**Table 5:** Ethnicity, socioeconomic indicators, and clinical outcomes.

	Non-Hispanic Black	Asian	Hispanic	Other	Non-Hispanic White
N = 6418	N = 1342	N = 315	N = 2712	N = 166	N = 1883
PRISM III ROM, (%)	0.63 (0.30–1.64)	0.63 (0.30–2.21)	0.63 (0.30–1.64)	0.63 (0.30–2.07)	0.51 (0.30–1.30) **
PICU LOS (days)	1.85 (0.92–4.67)	2 (0.86–4.92)	1.80 (0.92–4.23)	1.61 (0.88–3.89)	1.70 (0.85–3.95) *
Hospital LOS (days)	7.67 (3.5–17.7)	9.55 (4.2–19.9)	7.83 (3.8–15.7)	6.75 (3.6–18.6)	7.82 (3.8–16.1) ^NS^
Mortality, n (%)	47 (3.50)	14 (4.44)	76 (2.80)	7 (4.22)	67 (3.56) ^NS^
**N = 5912**	**N = 1246**	**N = 270**	**N = 2589**	**N = 140**	**N = 1667**
Poverty level (%)	18.6 (11–28)	11.4 (6.3–21)	22.1 (14–30)	16.1 (10–25)	12.3 (6.6–17) **
Median income/yr., USD	45.8 (37–62)	65.2 (43–92)	42.9 (34–58)	49.8 (39–65)	61.9 (45–84) **
Food insecurity (%)	21.6 (17–28)	16.6 (15–22)	17.7 (15–22)	18.4 (15–24)	16.7 (14–20) **

Values are median (IQR; 25th–75th). PRISM: Pediatric Risk of Mortality ROM: Risk of mortality; LOS, length of stay. Poverty percentage reflects the percentage of individuals in the patient’s zip code living in poverty; Median income in thousands of USD; Food Insecurity percentage reflects the percentage of individuals in the patient’s zip code living with food insecurity; * *p* < 0.05; ** *p* < 0.0001 by Kruskal–Wallis. Mortality analysis by Chi-square test. ^NS^ Non-significant.

**Table 6:** Insurance category, income, and food insecurity.

	Government Insurance	Non-Government Insurance	
	N = 3979	N = 1933	*p* Value
Median income/yr USD	43.9 (36–59)	64.7 (45–87)	<0.0001
Poverty level (%)	20.7 (13.2–28.8)	11.7 (6.3–18.3)	<0.0001
Food Insecurity (%)	18.8 (15.5–24)	16.1 (13.9–19)	<0.0001

Values are median (IQR; 25th–75th). Median income in thousands of USD; the poverty percentage reflects the percentage of individuals in the patient’s zip code living in poverty; Food Insecurity percentage reflects the percentage of individuals living with food insecurity in the patient’s zip code. Comparison by Mann–Whitney test.

**Table 7:** Prevalence of food insecurity by income category.

	Income Category			
	Low	Middle	High	
	N = 1501	N = 2956	N = 1455	*p* Value
% Food Insecurity	23.4 (18–29)	18.8 (16–22)	14.3 (13–16)	<0.0001 *

Values are median; IQR (25th–75th) and represent percentages of food insecurity according to income level. * *p* < 0.0001 by Kruskal–Wallis test.

## Data Availability

Data are contained within the article.
